# Long-term in vitro degradation and in vivo evaluation of resorbable bioceramics

**DOI:** 10.1007/s10856-020-06488-1

**Published:** 2021-01-21

**Authors:** Ying-Cen Chen, Pei-Yi Hsu, Wei-Hsing Tuan, Chih-Yi Chen, Chia-Jung Wu, Po-Liang Lai

**Affiliations:** 1 Department of Materials Science and Engineering, National Taiwan University, Taipei, Taiwan; 2 Department of Materials Science and Engineering, National Taiwan University of Science and Engineering, Taipei, Taiwan; 3 Department of Orthopedic Surgery, Bone and Joint Research Center, Chang Gung Memorial Hospital at Linkou, College of Medicine, Chang Gung University, Taoyuan, 333 Taiwan

## Abstract

An essential criterion for the selection of resorbable bioceramics is their ability to degrade inside human body within a reasonable time frame. Furthermore, if the bioceramic can release beneficial ions, such as strontium, as it degrades, recovery time might be shortened. The present study demonstrates that strontium-containing calcium sulfate (Sr,Ca)SO_4_ can fulfill these criteria. A long-term in vitro degradation analysis for 12 weeks using sintered (Sr,Ca)SO_4_ discs in phosphate buffered solution (PBS) was conducted. The sintered (Sr,Ca)SO_4_ disc was then implanted into defects in the distal femur of rats. The degradation rate of (Sr,Ca)SO_4_ discs showed a strong dependence on the Sr content. Similar results were observed between the long-term in vitro degradation analysis and the in vivo evaluation. The sintered (3.8%Sr,Ca)SO_4_ disc lost more than 80% of its initial weight after soaking in PBS with shaking at 37 °C for 12 weeks. After 12 weeks in vivo, the remaining volume of the (3.8%Sr,Ca)SO_4_ disc within the bone defect was ~25%. Over the same time period, new bone was formed at a relative volume of 40%. This study demonstrates the potential of (Sr,Ca)SO_4_ bioceramic, and the benefits of using a long-term degradation test during the evaluation of resorbable bioceramics.

## Introduction

The development of bioceramics is intended to reduce pain by shortening the healing time of patients; however, extensive in vitro and in vivo testing is required before bioceramics can become widely available. Due to the numerous tests, the development of novel materials for bone grafts is a costly and time-consuming process. However, many medical needs are still currently unmet. For example, the defect left behind after the removal of a musculoskeletal sarcoma is usually large [1]. The time needed to heal such large bone defects is at least 3–6 months. For patients with osteoporosis, the time needed is even longer [2]. Therefore, there is a need to develop bone grafts that can reduce the healing time for bone defects.

Bioceramics can generally be classified as resorbable or nonresorbable. While the degradation process occurs, new bone is formed and the defect is healed. Table 1 lists results from recent in vivo studies on the development of resorbable bioceramics [3–10]. Several observations can be made from this list:

1. Strontium (Sr) is known for its ability to enhance bone formation and reduce bone resorption [2–5, 7, 10]. In order to demonstrate the benefits of Sr-containing bioceramics, various studies using animal models have been performed. Though the defects introduced into animals are small in size, these in vivo studies all indicate that the addition of a small amount of Sr helps in bone regeneration and remodeling.
2. Due to reports of Sr having negative effects on the heart [http://www.ema.europa.eu/docs/en_GB/document_library/Medicine_QA/human/000560/WC500142021.pdf], the amount of Sr has to be low. From the reports shown in Table 1, the amount of Sr in these bioceramics is relatively low. Bioceramics such as calcium sulfate [3], calcium silicate [4], and calcium phosphate [5–10] act as carriers for the Sr ions. The degradation mechanisms and rates for bioceramics differ from one another [11–13], and the release behavior of Sr depends strongly on the degradation of bioceramic carriers in body fluid.
3. Bioceramics are used in the forms of paste [3], porous pellets [4–9, 11], or granules [10]. These forms increase the surface area of the implant in contact with body fluid.

**Table 1:** Summary of recent in vivo investigations of Sr-containing bone grafts

Bioceramic carrier/form	Sr content	Processing	Animal model/defect size/harvest time	Reported benefits	References
α-CaSO_4_ 2H_2_O or α-CaSO_4_ 1/2H_2_O/paste	10 mol%	Coprecipitation or hydrothermal	Tibia, rats/3 × 5 mm/4, 8, 12 weeks	Increased bone volume, bone mineral density	Li et al. [3]
CaSiO_3_/porous sintered disc	10 mol%	Coprecipitation/calcination	Calvarial, ovariectomy rats/ϕ 10–15 mm/4 weeks	Bone regeneration, angiogenesis	Lin et al. [4]
Sr_5_(PO_4_)_3_OH hydroxyapatite/porous sintered disc	59 wt%	Hydrothermal/calcination	Calvarial, rats/ϕ 5 mm/1, 3 months	Bone formation, bone remodel	Yang et al. [5], Pan et al. [6]
Calcium polyphosphate/porous sintered disc	1 wt%	Coprecipitation/calcination/quenching	Foreleg radius, rabbit/ϕ 15 mm/4, 8,16 weeks	Bone formation	Tian et al. [7], Chen et al. [8], Qiu et al. [9]
23%HAp + 77% TCP/granule	5 wt%	Precipitation/heat treatment	Femoral neck, ovariectomy rabbit/5 × 4 mm/12 weeks	Bone remodeling	Zarins et al. [10]
CaSO_4_/sintered disc	1, 5, 10 wt%	Solid-state reaction	Distal femur, rats/3 × 4 mm/12 weeks	Bone formation	This study

*HAp* hydroxyapatite, *TCP* tricalcium phosphate

The transition from in vitro degradation analyses to in vivo evaluation is a time-consuming process. However, a thorough in vitro characterization is crucial to the in vivo evaluation. In this study, the degradation behavior of Sr-containing calcium sulfate (Sr-CaSO_4_) was analyzed over a long period (12 weeks). Results were then confirmed using an in vivo investigation in rats.

## Experimental procedures

### Preparation and characterization of specimens

A solid-state reaction was used to prepare the Sr,Ca)SO_4_ powders. The raw powders used in the present study were calcium sulfate hemihydrate (CaSO_4_•1/2H_2_O, JT Baker, USA) and Sr sulfate (SrSO_4_, Alfa Aesar, USA) powders. The weight ratio of SrSO_4_ to CaSO_4_•1/2H_2_O was 1, 5, and 10 wt% (Table 2). These compositions resulted in 0.74, 3.8, and 7.6 mol% Sr in the (Sr,Ca)SO_4_ solid solution, assuming the SrSO_4_ was completely dissolved into the CaSO_4_. The two powders were mixed in a turbo mixer in a milling media of ethyl alcohol and zirconia balls for 4 h. After milling, the slurry was dried first with an evaporator and then an oven at 100 °C for 12 h.

**Table 2:** Characteristics of the sintered discs used for in vitro and in vivo evaluations

SrSO_4_ in starting powder/wt%	Crystalline phase	Relative density/%	Grain size/μm	Notation
0	CaSO_4_	93.9% ± 0.8%	29	CS
1	(0.74%Sr, Ca)SO_4_	94.3% ± 0.3%	23	0.74%Sr
5	(3.8%Sr, Ca)SO_4_	93.1% ± 0.4%	19	3.8%Sr
10	(7.6%Sr, Ca)SO_4_	93.5% ± 0.3%	20	7.6%Sr
100	SrSO_4_	84.0% ± 1.4%	4	Sr

The dried lumps were crushed with a pestle and mortar. The resulting powder was sieved with a #150 mesh plastic sieve before use. The opening of the mesh was 103 μm. This sieving step could remove large agglomerates to ensure the uniformity of green body. Disc-shaped specimens were prepared by die-pressing, then sintered at 1100 °C for 1 h. The specimen dimensions for in vitro evaluations were 8.5 mm in diameter and 2.9 mm in height. For the in vivo evaluations, the dimensions were 3.0 mm in diameter and 4.0 mm in height. Pure CaSO_4_ and SrSO_4_ specimens were also prepared using the same procedures. The density of the specimens was estimated by measuring their weight and dimensions. The theoretical densities used for calcium sulfate and Sr sulfate were 2.96 [14] and 3.96 g/cm^3^ [15], respectively. Based on the CaSO_4_–SrSO_4_ phase diagram proposed by Bushuev et al. [16] and later confirmed by Chen et al. [17], an amount lower than 10 mol% of SrSO_4_ could dissolve completely into CaSO_4_ during sintering at 1100 °C. Since the crystalline structure of CaSO_4_ and SrSO_4_ was the same [14, 15], the Sr ions replaced Ca ions during sintering [16, 17]. The theoretical density of the (Sr,Ca)SO_4_ specimen discs could thus be estimated using the theoretical densities for CaSO_4_ and SrSO_4_. The relative density of the specimen was calculated by dividing the density to the theoretical density.

The cross section of the specimen disc was exposed by grinding with SiC paper. The grain boundaries were revealed by heating at 1000 °C for 0.5 h. More than 200 grains were counted for measurements of the average grain size. For the phase analysis, the sintered disc was crushed into a powder first. X-ray diffraction (XRD) of the powder was then applied using X-rays generated from the synchrotron located at Hsinchu, Taiwan.

### In vitro evaluation

**Fig. 1:**
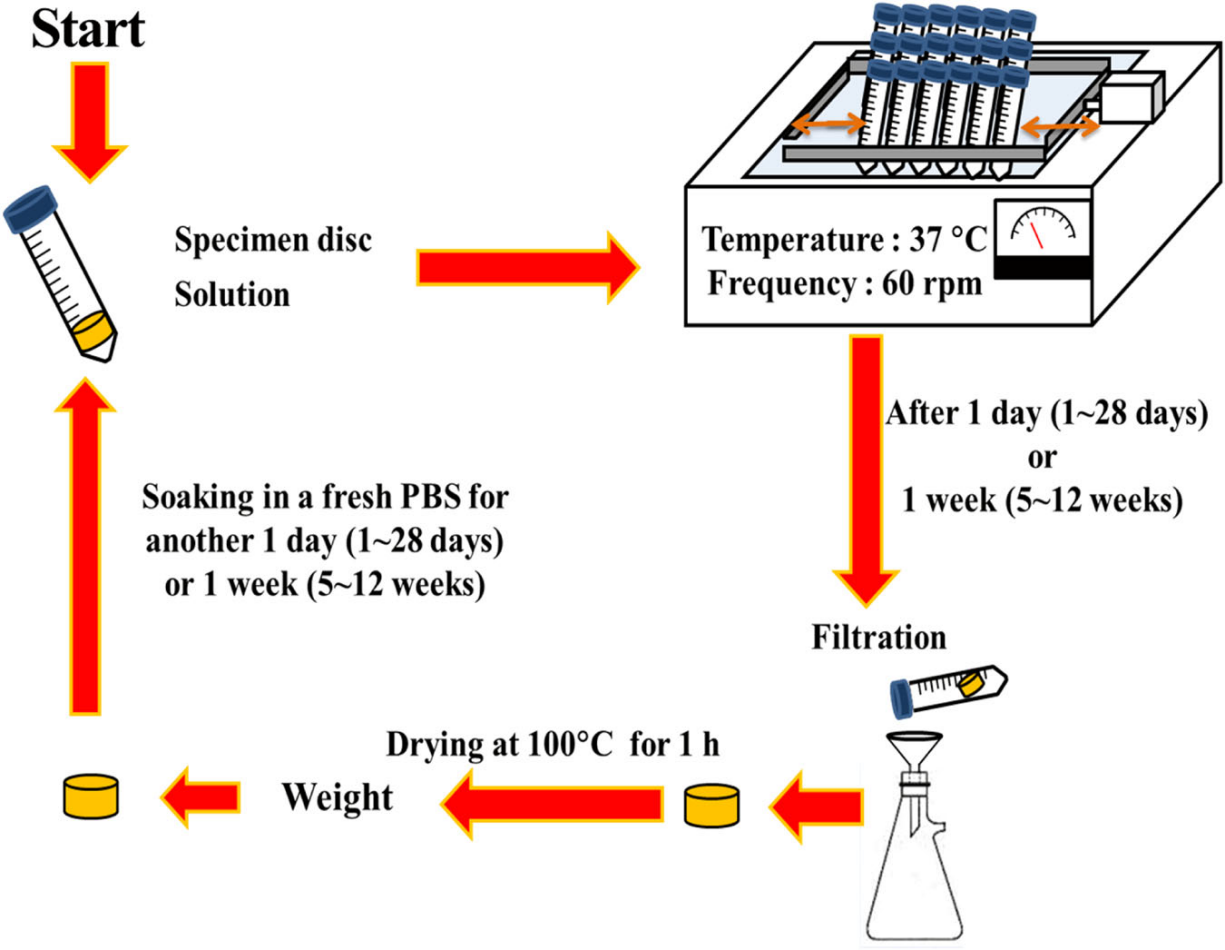
Schematic showing the procedures for the long-term degradation test

The present study focused on the degradation behavior of bioceramics in fluid. A time span chosen for this in vitro evaluation was 12 weeks, the schematic to illustrate the testing procedures is shown in Fig. 1. The 0.5 g sintered disc was soaked in a phosphate buffered solution (PBS, Gibco Co., USA) in a test tube. The initial amount of PBS was 5 ml and was adjusted according to the residual weight of the specimen disc to maintain a constant ratio of 1 (g disc) to 10 (ml of solution) throughout the degradation test. The test tubes were left in a water bath at 37 °C for 24 h. The tubes were shaken in the water bath at a frequency of 60 rpm to simulate the dynamic environment within the body.

After 24 h, the disc was separated from the solution using filtration paper. The specimen was dried on the filtration paper at 100 °C for 1 h and the residual weight was then measured. The dried specimen disc was then put into another test tube containing fresh PBS solution. The process was then repeated for 28 days. After 28 days, the ratio of specimen weight to PBS volume was changed to 1:70. This ratio was then kept constant from weeks 5 to 12, refreshing the PBS every week.

### In vivo evaluation

The in vivo test adhered to the regulations of the Animal Institutional Review Board of Chung Gung Memorial Hospital with an approval number of IACUC 2016092004. Defects with a diameter of 3 mm and depth of 4 mm were introduced into both distal femurs in Sprague Dawley rats. Seventeen rats were used for this study. The sterilization for sintered disc was carried out in an autoclave at 121 °C for 30 min. The pressure applied was 1.2 kg/cm^2^. The rats recovered well after the surgery. The defects were monitored via X-ray imaging at 1, 4, and 12 weeks post surgery. The femur was harvested at week 12 and micro-computed tomography (micro-CT, NanoSPECT/CT, Mediso Co., Hungary, X-ray: 55 KeV, 980 μA, exposure time: 170 ms) was used to estimate the volume of the remaining implant.

For histology observation, the distal femur was kept in 10% neutral buffered formalin for 1–2 days. The specimen was then decalcified (DECALCIFIER II, Surgipath Medical Ind., Richmond, USA) and dehydrated in ethanol (first 30 or 50%, then 70, 85, 95, and 100%). In order to make sure that the sintered specimen could be decalcified, a preliminary test was conducted, during which a sintered CaSO_4_ specimen disc completely disappeared after decalcification. The decalcified and dehydrated femur was mounted in paraffin and cut into sections with a thickness of 4–5 μm. Two stains, hematoxylin & eosin and Masson’s trichrome (ArrayBiotech Co., Taiwan), were used to reveal bone and marrow. The area of new bone was estimated using ImageJ-3 from stained images. Statistical analysis was carried out using a one-way ANOVA test.

## Results

### Characterization of specimens

**Fig. 2:**
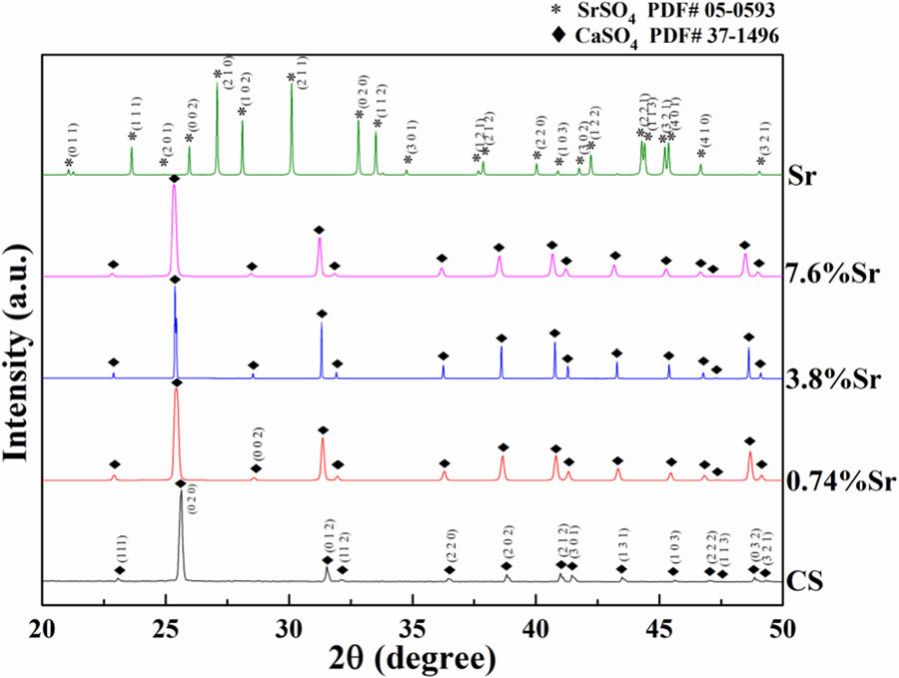
Synchrotron X-ray diffraction patterns for sintered specimens

Figure 2 shows the synchrotron XRD patterns for the sintered (Sr,Ca)SO_4_ specimens. As the starting amount of SrSO_4_ was lower than 10 wt%, there was no SrSO_4_ peak detected after sintering at 1100 °C. The crystalline phase for sintered (Sr,Ca)SO_4_ specimen was the same as that of anhydrite CaSO_4_, a hexagonal structure (PDF # 37-1496). These CaSO_4_ peaks shifted to their left slightly. Taking the (020) peak as the example, the peak shifts by 0.19 (0.73%Sr), 0.24 (3.8%Sr), and 0.27 (7.6%Sr) 2θ, respectively. It suggests the dissolution of SrSO_4_ into CaSO_4_ during sintering. Furthermore, the solution of Sr into CaSO_4_ increases with the increase of Sr content. Since the valence states of Ca and Sr were the same, the crystalline structures of SrSO_4_ and CaSO_4_ were also the same and the solubility of SrSO_4_ in CaSO_4_ is therefore high [16, 17].

**Fig. 3:**
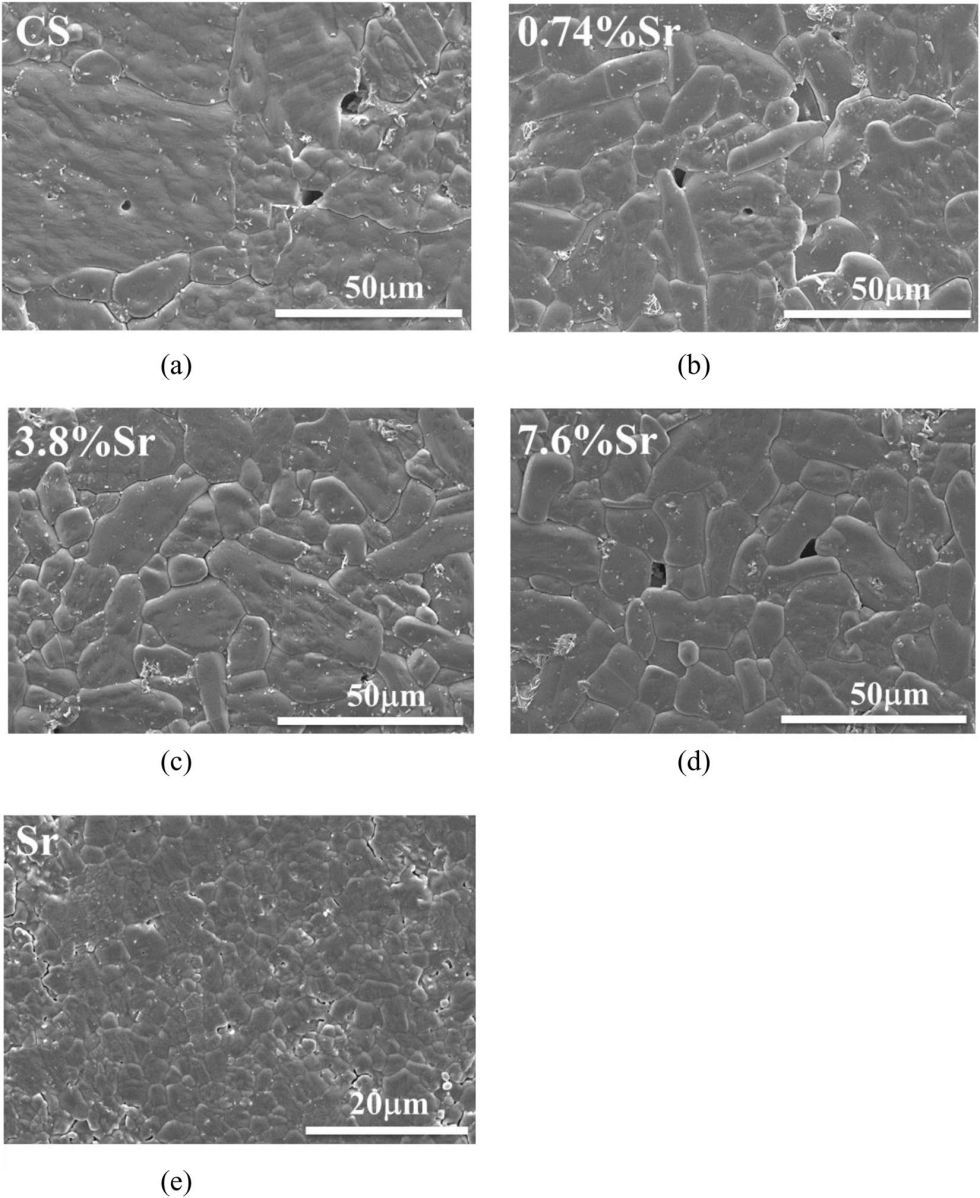
Typical micrographs for sintered **a** CaSO_4_ (CS), **b** (0.74%Sr,Ca)SO_4_ (0.74%Sr), **c** (3.8% Sr,Ca)SO_4_ (3.8%Sr), **d** (7.6% Sr,Ca)SO_4_ (7.6%Sr), and **e** SrSO_4_ (Sr) specimens

After sintering at 1100 °C, the relative density of the sintered CaSO_4_ and (Sr,Ca)SO_4_ specimens was higher than 93% (Table 2), whereas the density of the sintered SrSO_4_ specimen was lower than 85%. Figure 3 shows typical scanning electron micrographs for the cross-sections of sintered specimens. After counting more than 200 grains, the average grain size in the pure CaSO_4_ specimen was 29 μm (Fig. 3a). After the addition of 3.8 or 7.6 mol% Sr, the size of CaSO_4_ grains was reduced to around 20 μm. The grains in the SrSO_4_ specimen were relatively small at 4 μm (Fig. 3e). Apart from SrSO_4_ specimen, Table 2 indicated that the CaSO_4_ and (Sr,Ca)SO_4_ specimens have reached their final stage of sintering. The pores are likely isolated to each other. It can be confirmed by the microstructure observation (Fig. 3).

### In vitro evaluation of bioceramic degradation

**Fig. 4:**
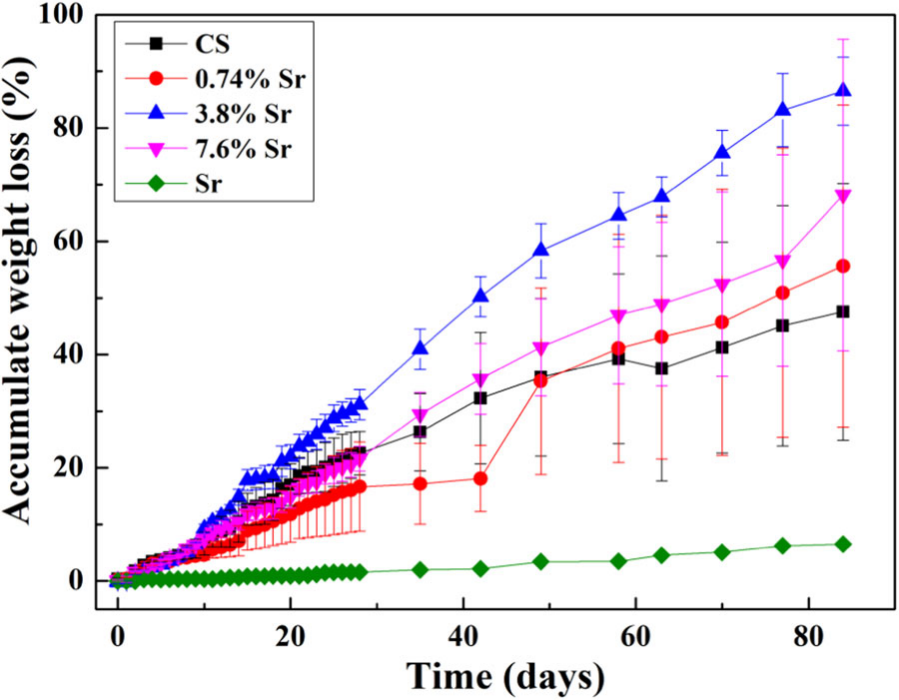
Cumulative weight loss for the sintered discs for 28 days in PBS. The PBS was refreshed every day; the weight was monitored on a daily basis

The weight of the specimen discs was monitored before and after soaking in PBS for 12 weeks in vitro to monitor degradation. For the first 28 days, the PBS was refreshed every day. Figure 4 shows the cumulative weight loss of sintered specimens as a function of time. After 28 days, the cumulative weight loss of CaSO_4_ specimen reached a value of 23%. For the (3.8%Sr,Ca)SO_4_ specimen, the weight loss was 31%. The sintered SrSO_4_ specimen showed little weight loss (i.e., less than 2%) after 28 days in PBS.

**Fig. 5:**
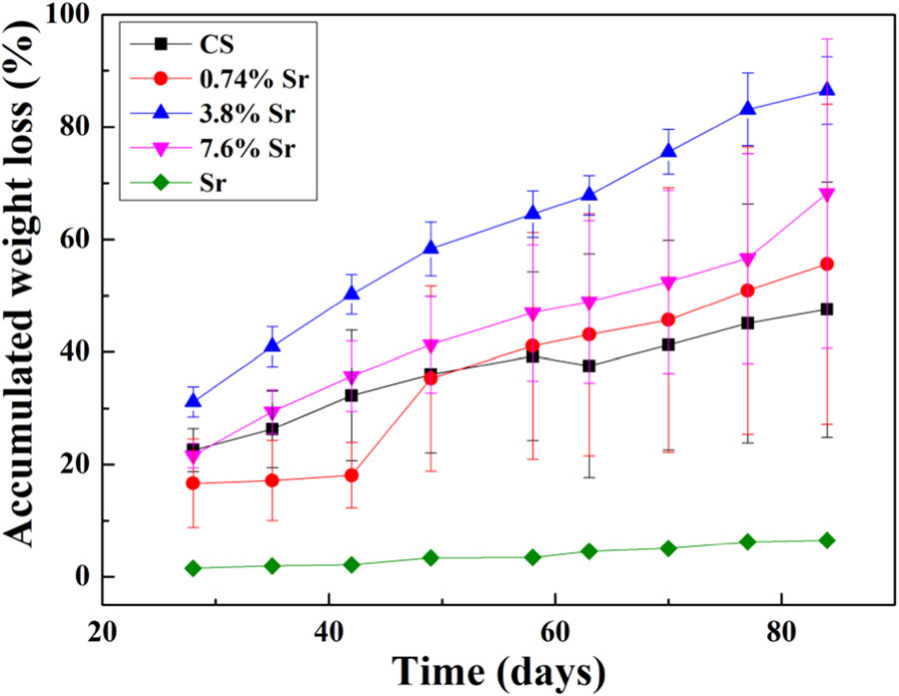
Cumulative weight loss for the sintered discs in PBS from week 5 to week 12. The PBS was refreshed every week; the weight was monitored on a weekly basis

Few in vitro studies continue degradation testing beyond 28 days [12, 18, 19]. However, bone defects typically take much longer to heal so the degradation behavior should be monitored over a much longer time period. Thus, our study carried out degradation testing for 12 weeks (Fig. 5). The addition of 1 wt% SrSO_4_ reduced the weight loss rate of CaSO_4_ during the first 6 weeks (Fig. 5); however, this rate increased from weeks 7 to 12. The cumulative weight loss for the sintered (3.8%Sr,Ca)SO_4_ disc was the highest at 87% after 12 weeks. For the same time span, the cumulative weight loss for the SrSO_4_ specimens was only 6.5%.

**Fig. 6:**
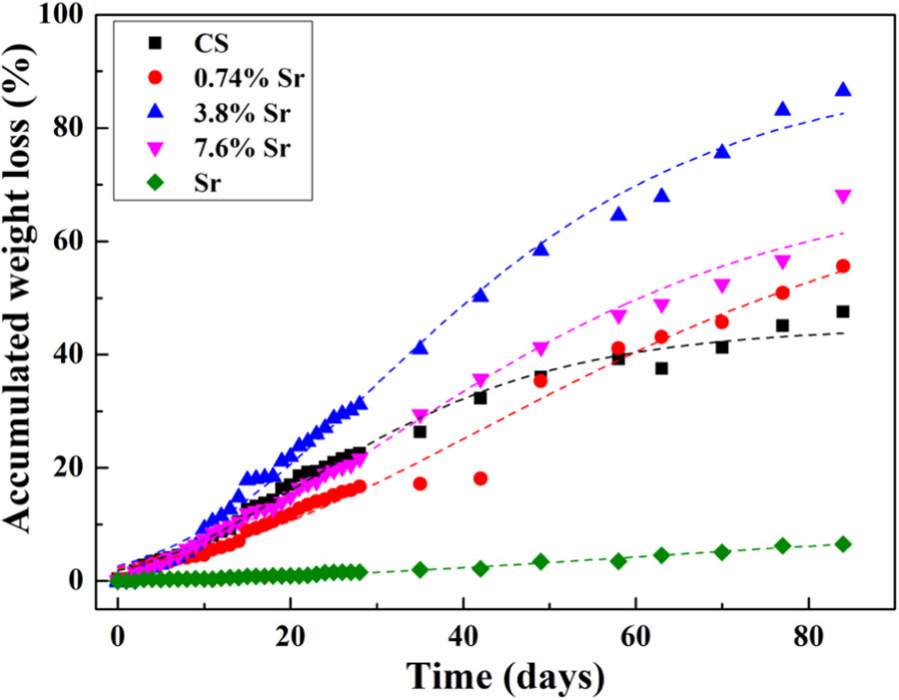
Cumulative weight loss for the sintered discs in PBS. The weight change was monitored on a daily basis in the first 4 weeks, then on a weekly basis from weeks 5 to 12

Since the PBS was refreshed every day for the first 28 days and every week thereafter, the influence of soaking time on the degradation behavior could be investigated (Fig. 6). The weight loss of (Sr,Ca)SO_4_ specimens was larger than that of CaSO_4_ specimens. Furthermore, the weight loss showed a parabolic relationship with time, suggesting that the rate of weight loss decreases over time (Tables 3 and 4).

**Table 3:** Residual weight of sintered discs in PBS after 12 weeks

Specimen	Residual weight/%
CS	53 ± 23
0.74%Sr	44 ± 28
3.8%Sr	14 ± 6
7.6%Sr	32 ± 28
Sr	94 ± 0.4

**Table 4:** Residual volume of the (Sr,Ca)SO_4_ sintered disc at the rat distal femur after implantation for 12 weeks

Specimen	Residual volume of sintered disc/vol %	Volume of new bone/vol%
Defect	–	8 ± 3
CS	39 ± 9	20 ± 9
0.74%Sr	31 ± 12	20 ± 7
3.8%Sr	27 ± 4	41 ± 4
7.6%Sr	37 ± 7	24 ± 5

The volume of new bone is also shown. The ratio is expressed in terms of original volume of the sintered disc

### In vivo evaluation

The in vitro evaluation over a time span of 12 weeks indicated that the degradation of sintered SrSO_4_ was too slow in the context of bone healing and thus, would not be a suitable resorbable ceramic due to its low degradation rate (Fig. 6). An in vivo evaluation of the SrSO_4_ specimen was thus not performed.

**Fig. 7:**
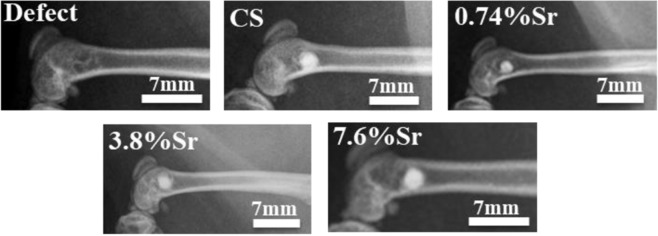
X-ray images for the sintered (Sr,Ca)SO_4_ discs in rat distal femurs 12 weeks after operation

The healing of bone defects at the distal femur was monitored with X-ray imaging at 1, 4, and 12 weeks post surgery. The X-ray images at week 12 are shown in Fig. 7. Since the density of sintered CaSO_4_ or (Sr,Ca)SO_4_ discs is much higher than the neighboring bone (>90% relative density), the radiopacity of the remaining sintered disc was high. The X-ray images indicated that the sintered discs remained within the bone defect after 12 weeks.

**Fig. 8:**
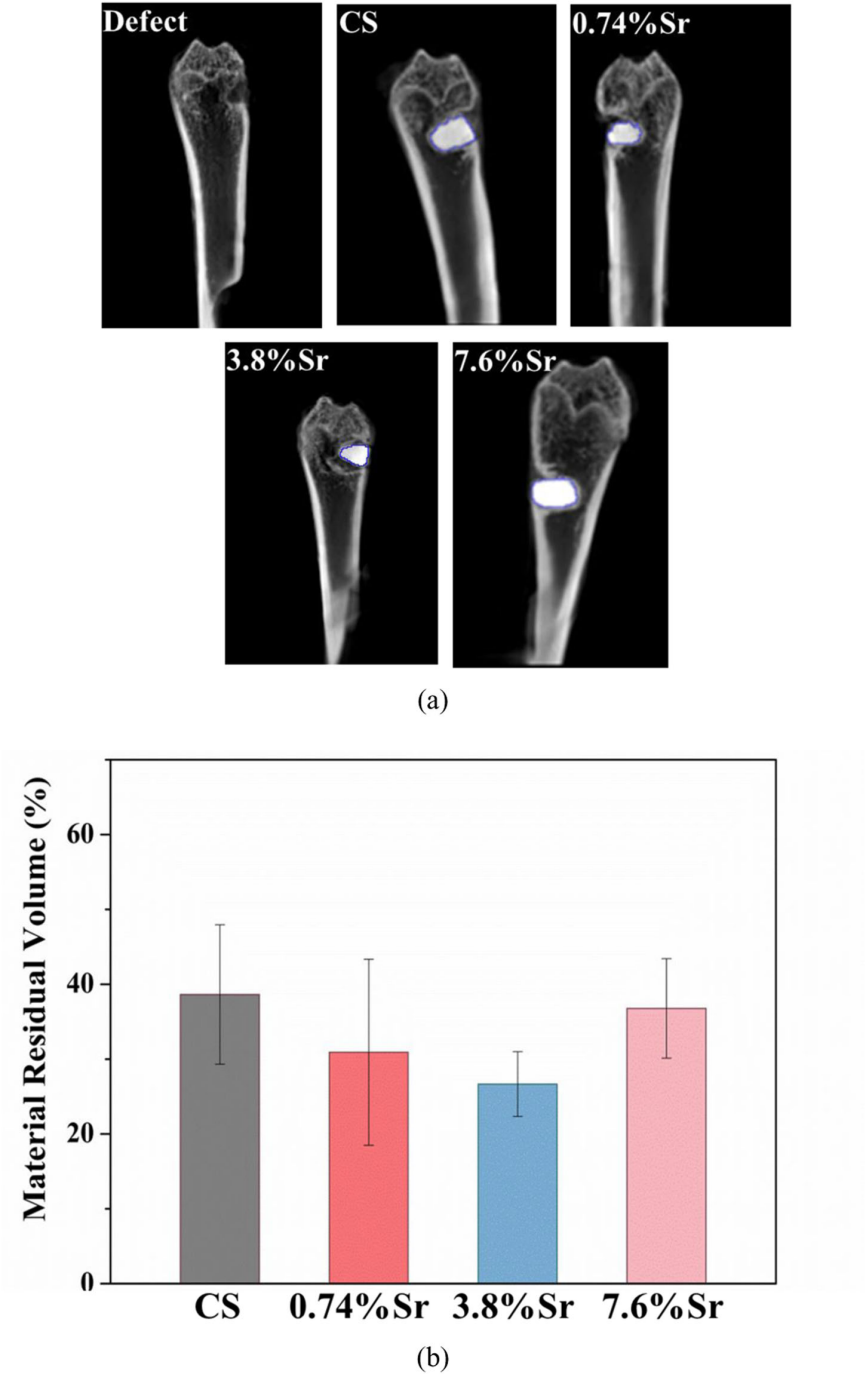
**a** Micro-CT images for the sintered (Sr,Ca)SO_4_ discs in rat distal femurs at 12 weeks. **b** Volume of remaining disc after 12 weeks

The micro-CT images could also detect the remains of the sintered discs at the bone defect after week 12 (Fig. 8a). The contrast for the residual sintered disc was clear enough to estimate its area in each image. The micro-CT images were collected to encompass the whole disc edge to edge and the area of the remaining specimen in each image was estimated. By summing up the total area for one specimen, the volume of the remaining disc was determined (Fig. 8b). The residual volumes were around 20–40%, depending on the material. The statistical analysis showed no significant difference between sintered CaSO_4_ and (Sr,Ca)SO_4_ specimens.

**Fig. 9:**
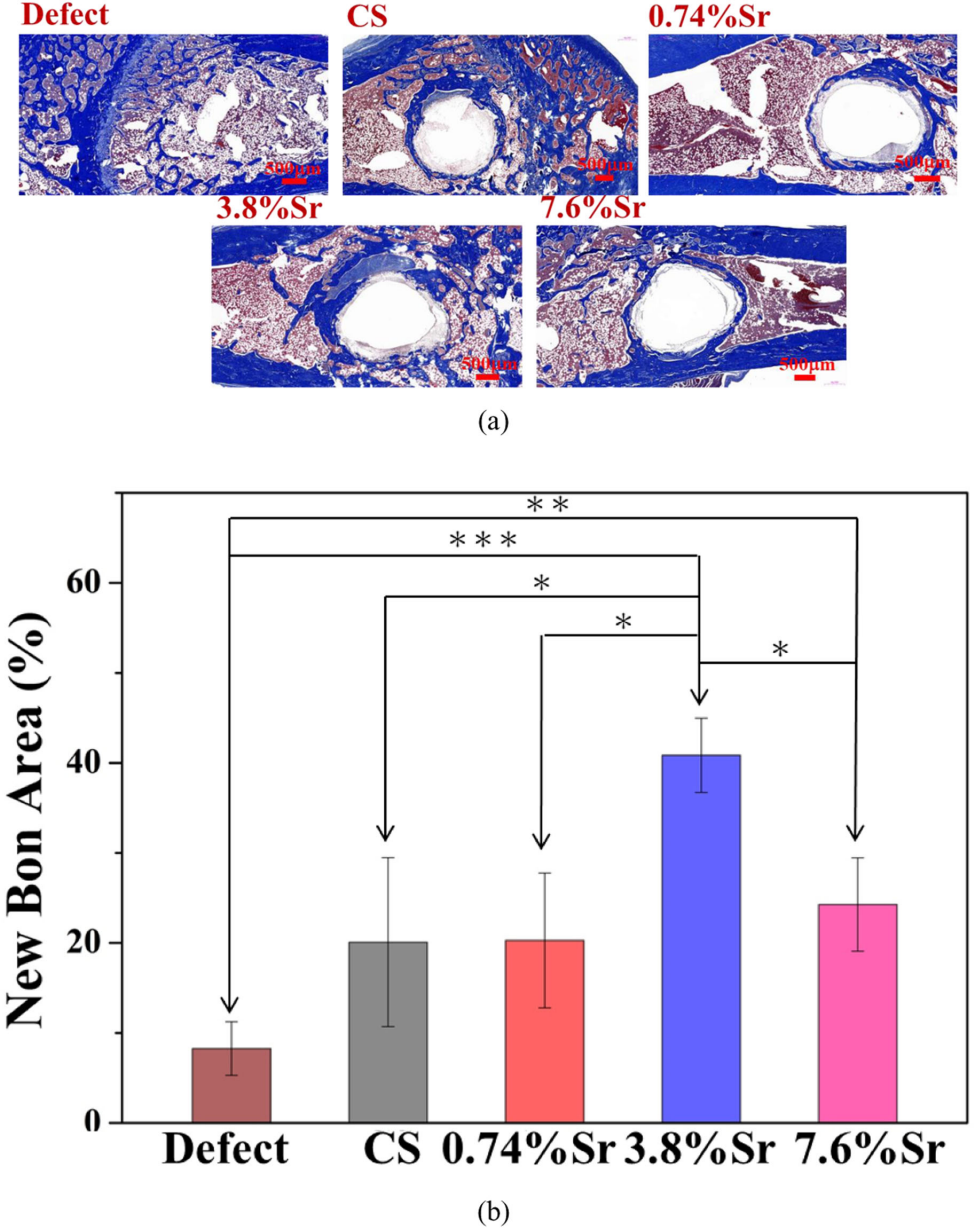
**a** Histology of sintered (Sr,Ca)SO_4_ discs in rat distal femurs at 12 weeks. **b** The area fraction of new bone in the image (*P < 0.05, **P < 0.01, and ***P < 0.001)

Histological staining for each sintered disc after 12 weeks is shown in Fig. 9a. The remains of each specimen were removed during the decalcification treatment. The new bone was mainly localized near the interface area (blue in Masson’s trichrome stain), which differs from previous reports showing bone growing into porous bodies [4–10]. The amount of new bone can thus be estimated with high confidence (Fig. 9b). The volume of new bone was lowest in the control group (defects only). Confirmed by statistical analysis, the sintered (3.8%Sr,Ca)SO_4_ disc resulted in the greatest formation of new bone among all (Sr,Ca)SO_4_ specimens.

## Discussion

Resorbable bioceramics have been developed with the goal of shortening the healing time for patients with bone defects. Bone grafting by surgery is the last resort for any patient; nevertheless, it is a valuable chance to help avoid any future malunion or nonunion after healing. Therefore, the present study proposes an idea for the design of a resorbable bone graft and the value of a long-term degradation test.

### Design of resorbable bioceramics

Table 1 shows several options for resorbable bioceramics. In order to compare these bioceramics, the following chemical and physical aspects have to be taken into account:

1. Chemical aspects: resorbable bioceramics are mainly calcium salts, such as calcium sulfate, calcium silicate, calcium phosphate, and their combinations [3–10]. These calcium salts can release Ca ions during degradation. The release of Ca ions aids in the recovery of bone defects [3], other ions such as Sr, Si, and Ce are also helpful for the formation of bone [2–10, 20]. Resorbable bioceramic implants can thus be thought of as ion carriers. The carrier should not only release Ca ions, but also beneficial ions, such as Sr ions, within the bone defect.
2. Physical aspects: resorbable ceramics come in different physical forms. They can be porous blocks, granules, or paste [3–10]. It is well accepted that the pore size is critical for angiogenesis and the ingrowth of new bone. Nevertheless, a consensus on the optimal amount of pores and their size within bioceramics is still lacking [13].

The bioceramics evaluated in this study can be divided into three groups: CaSO_4_, Sr-CaSO_4_, and SrSO_4_. Results from the comprehensive characterization of density, grain size, and crystalline phase of these ceramics can be seen in Table 2.

Calcium sulfate hemihydrate has been used as a bone graft material for more than 100 years, its biocompatibility is well accepted [3]. Nevertheless, the degradation rate of this material is too fast, complete degradation can take place within a week [21]. The degradation of calcium sulfate hemihydrate discs may be even more unpredictable. Hsu et al. applied a sintering technique to remove the pores and crystal water from the calcium sulfate hemihydrate structure, resulting in a significant reduction in the material’s degradation rate [12]. As demonstrated in the present study, the sintered CaSO_4_ specimen degraded 23% after 28 days, then another 25% from weeks 5 to 12 (Fig. 6). Around half the weight was left after the 12-week in vitro test. For the in vivo test, about 35% volume remained within the bone defect after 12 weeks, with 20% new bone being formed during the same time span. This demonstrates that the sintered CaSO_4_ specimen may be a suitable bone graft material.

This study also investigated an Sr-containing group, (Sr,Ca)SO_4_. The density of sintered (Sr,Ca)SO_4_ was close to that of the sintered CaSO_4_ specimen (Table 2); nevertheless, the grain size was smaller. Microstructure analysis (Fig. 3) demonstrated that the Sr solutes are likely precipitated at the boundaries of CaSO_4_ grains. The size of CaSO_4_ grains in the (Sr,Ca)SO_4_ specimens was thus smaller. The degradation rate of (Sr,Ca)SO_4_ was faster than that of the pure CaSO_4_ specimen. Furthermore, the degradation rate also shows dependence on Sr content (Figs. 4–6).

The degradation of SrSO_4_ was only 6% in the time span of 12 weeks, which is too slow in terms of degradation rate for bone healing. The sintered SrSO_4_ was therefore not used in the in vivo study. As such, a long-term degradation test can serve as a screening test for choosing the appropriate resorbable bioceramic.

### Value of long-term degradation test

Table 1 summarizes the in vivo studies on several Sr-containing bioceramics [3–10]. Different from previous studies, the specimens used in the present study were prepared by sintering at elevated temperature (1100 °C), and featured a small amount of isolated pores. In this case, degradation only takes place from the specimen surface. Since the size and shape of specimens were the same, the only factor to affect the degradation rate was the specimen composition. The use of such sintered discs gives us chance to evaluate the effect of Sr content. The degradation of calcium sulfate generates calcium phosphate at the surface during degradation [12]. Therefore, the degradation rate decreases over time due to the change from calcium sulfate to calcium phosphate on the surface.

Since there are no open pores in the sintered (Sr,Ca)SO_4_ specimen, new bone can only be formed at the interface between residual material and the surrounding bone tissue. Some inflammatory cells and soft tissue were found on the surface of residual specimens (Fig. 9b), indicating the formation of a soft callus first, followed by new calcified bone. This suggests that the healing of bone defects likely involves three overlapping stages: the early inflammatory, recovery, and remodeling stages [13].

**Fig. 10:**
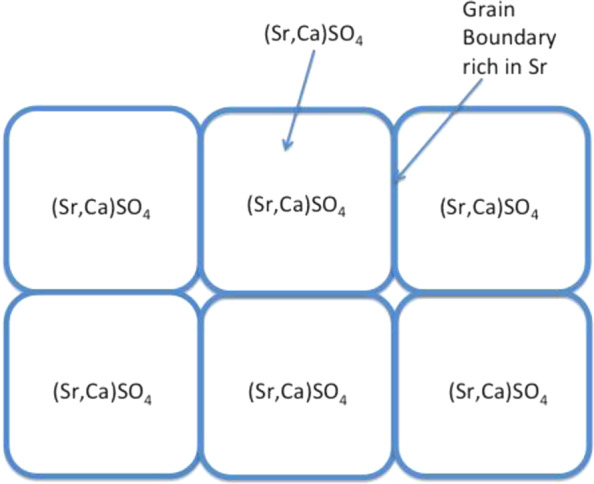
Schematic for the sintered (Sr,Ca)SO_4_ specimen. The pores within the specimen are isolated to each other. The degradation of sintered specimen is contributed from the dissolution of (Sr,Ca)SO_4_ grains and their boundaries

The weight loss rate for the (3.8%Sr,Ca)SO_4_ in PBS was the highest (Fig. 6) and similarly, its residual volume within the bone defect was the smallest (Fig. 8a). More importantly, the volume of new bone was the largest (Fig. 9). A schematic for the sintered specimen is shown in Fig. 10. For the (Sr,Ca)SO_4_ grains, the addition of Sr increases its lattice parameter, as demonstrated by the peak shift in the XRD patterns (Fig. 2). The increase of lattice volume enhances the release of Ca and Sr ions from (Sr,Ca)SO_4_ lattice. The boundary between (Sr,Ca)SO_4_ grains is likely rich in Sr ions. Such segregation may affect the release of ions from grain boundaries. Based on the in vitro weight loss evaluation (Figs. 4–6), the release from grain boundaries is likely reduced. As resulted from the competition between the dissolutiom from grain and from grain boundaries, a higher weight loss is observed for the sintered (3.7%Sr,Ca)SO_4_ disc, and a lower weight loss for the sintered CaSO_4_, (0.74%Sr,Ca)SO_4_, and (7.6%Sr,Ca)SO_4_ specimens. The present study indicates that the Sr content affects a lot the degradation rate of CaSO_4_. It can be related, at least partly, to the change of microstructure.

## Conclusions

As bone healing for defects lasts for a long time, so does the pain experienced by patients. The implantation of resorbable bioceramic materials could shorten this healing time and thus reduce pain. In the present study, a long-term in vitro degradation analysis is used to evaluate resorbable bioceramics. Results of the degradation test were supported by the in vivo evaluation in a rat distal femur model. Degradation times in PBS solution (in vitro) and in femur defects (in vivo) were very similar. The suitability of the Sr-containing CaSO_4_ specimen was validated with the long-term degradation test. This long-term degradation analysis showed that the addition of Sr into CaSO_4_ enhances its degradation rate. The degradation rate of CaSO_4_ was nearly doubled after the addition of 3.8 mol% Sr into CaSO_4_. This enhancement may be related to the modification of microstructure. The degradation of (Sr,Ca)SO_4_ specimens released both Ca and Sr ions, which promote the formation of new bone. The potential of using (Sr,Ca)SO_4_ as a bone graft material is therefore high.
